# Genome analysis of the ubiquitous boxwood pathogen *Pseudonectria foliicola*

**DOI:** 10.7717/peerj.5401

**Published:** 2018-08-24

**Authors:** Yazmín Rivera, Catalina Salgado-Salazar, Daniel Veltri, Martha Malapi-Wight, Jo Anne Crouch

**Affiliations:** 1 Mycology and Nematology Genetic Diversity and Biology Laboratory, US Department of Agriculture, Agriculture Research Service (USDA-ARS), Beltsville, MD, United States of America; 2 Department of Plant Biology and Pathology, Rutgers, The State University of New Jersey, New Brunswick, NJ, United States of America; 3 ARS Research Participation Program, Oak Ridge Institute for Science and Education, Oak Ridge, TN, United States of America; 4 Current affiliation: Center for Plant Health, Science and Technology, USDA, Animal and Plant Health Inspection Service, Beltsville, MD, United States of America; 5 Current affiliation: Bioinformatics and Computational Biosciences Branch, Office of Cyber Infrastructure and Computational Biology, National Institute of Allergy and Infectious Diseases, NIH, Rockville, MD, United States of America; 6 Current affiliation: Plant Germplasm Quarantine Program, USDA, Animal and Plant Health Inspection Service, Beltsville, MD, United States of America

**Keywords:** Volutella blight, Boxwood, Comparative genomics, Nectriaceae

## Abstract

Boxwood (*Buxus* spp.) are broad-leaved, evergreen landscape plants valued for their longevity and ornamental qualities. Volutella leaf and stem blight, caused by the ascomycete fungi * Pseudonectria foliicola and P. buxi*, is one of the major diseases affecting the health and ornamental qualities of boxwood. Although this disease is less severe than boxwood blight caused by * Calonectria pseudonaviculata* and * C. henricotiae*, its widespread occurrence and disfiguring symptoms have caused substantial economic losses to the ornamental industry. In this study, we sequenced the genome of * P. foliicola* isolate ATCC13545 using Illumina technology and compared it to other publicly available fungal pathogen genomes to better understand the biology of this organism. A * de novo* assembly estimated the genome size of * P. foliicola* at 28.7 Mb (425 contigs; N50 = 184,987 bp; avg. coverage 188×), with just 9,272 protein-coding genes. To our knowledge, * P. foliicola* has the smallest known genome within the Nectriaceae. Consistent with the small size of the genome, the secretome, CAzyme and secondary metabolite profiles of this fungus are reduced relative to two other surveyed Nectriaceae fungal genomes: * Dactylonectria macrodidyma* JAC15-245 and * Fusarium graminearum* Ph-1. Interestingly, a large cohort of genes associated with reduced virulence and loss of pathogenicity was identified from the * P. foliicola* dataset. These data are consistent with the latest observations by plant pathologists that * P. buxi* and most likely * P. foliicola,* are opportunistic, latent pathogens that prey upon weak and stressed boxwood plants.

## Introduction

Ascomycete fungi inhabit almost all known ecosystems, and play important roles as plant and insect pathogens, endophytes, mycoparasites, and saprobes (Arnold et al., 2009). Within the Ascomycota, the Nectriaceae family includes 55 genera with approximately 900 species (http://www.indexfungorum.org). Although best known as soil-borne saprobes or weak plant pathogens, several species in this family are responsible for extensive economic losses due to damage incurred to crops or in natural ecosystems (Halleen, Fourie & Crous, 2006; Malapi-Wight et al., 2016a; Windels, 2000). The systematics and taxonomy of the Nectriaceae family has been extensively studied (e.g., Salgado-Salazar et al., 2014; Lombard et al., 2015) however, outside of the genus *Fusarium*, only a small number of fungal species in this family have genome resources publicly available, including *Calonectria pseudonaviculata*, *C. pseudoteaudii*, *Dactylonectria macrodidyma* and *Ilyonectria destructans* (http://genome.jgi.doe.gov/Ilysp1/Ilysp1.home.html; Malapi-Wight et al., 2015; Malapi-Wight et al., 2016b; Ye et al., 2017). Whole genome resources are now commonly used to understand evolutionary characteristics of pathogenicity across fungi with different lifestyles (Lo Presti et al., 2015) and could become useful for the characterization and biosecurity analysis of undescribed pathogens (McTaggart et al., 2016).

**Figure 1:**
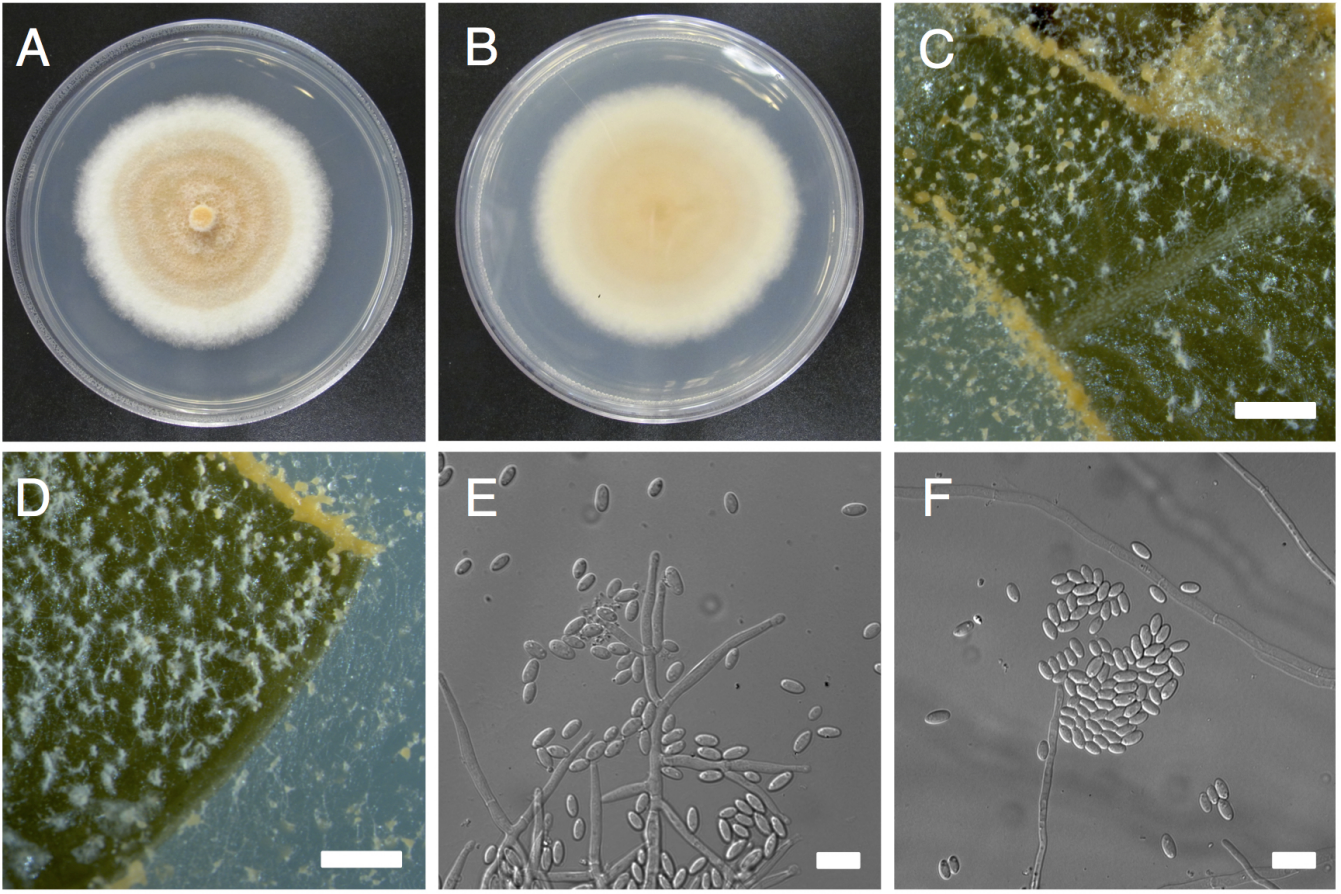
Morphological characters of *Pseudonectria foliicola* (ATCC 1354). (A) and (B) Mycelial growth on potato dextrose agar (A, front; B, back). (C) and (D) Sporulation on infected boxwood leaf tissue. E, Conidiophores. (E) and (F) conidia. Scale bars: B–D = 50 µm, E–F = 10 µm.

*Pseudonectria foliicola* and *P. buxi* (the latter formerly known as *Volutella buxi* or *P. rousseliana*) are nectriaceous species causing a ubiquitous leaf and stem blight disease on boxwood (*Buxus* spp.), known as volutella blight (Fig. 1). To date, this disease has been reported worldwide, throughout the US, Armenia, Belgium, Bulgaria Canada, China, Greece, Portugal, Spain, UK and Ukraine, among others (Farr & Rossman, 2018), although its distribution may extend further along with the distribution of boxwood plants. Infected plants may lack any disease symptoms, or they may manifest visually discernable physiological changes such as leaf discoloration, stem dieback and extensive pink fungal sporulation on the surface of leaves and twigs (Shi & Hsiang, 2014). The causal agent of volutella blight has been described as the species *P. buxi* (and synonyms) since the early nineteenth century. However, on the basis of morphological and molecular distinctiveness, Lombard et al. (2015) recently described *P. foliicola* as a second species of *Pseudonectria* that infects boxwood in New Zealand and the US. It is currently unclear to what extent previous sightings of volutella blight prior to the discovery of *P. foliicola* were actually caused by *P. buxi, P. foliicola,* or both of these pathogens.

The pathogens responsible for volutella blight disease have long been considered saprophytes or secondary invaders, however, recent studies by Shi & Hsiang (2014) identified *P. buxi* causing primary infection on wounded tissue contributing to boxwood decline. Reports from China and Italy confirm the impact of *P. buxi* as a primary pathogen of *Buxus* spp. (Shi & Hsiang, 2014; Garibaldi et al., 2016). Unlike boxwood blight disease caused by *C. pseudonaviculata* and *C. henricotiae* (Gehesquière et al., 2016), volutella blight primarily affects the ornamental value of boxwood, as plants are typically not killed by the fungal infection. Nonetheless, financial losses due to volutella blight may be considerable. For example, in 2008, economic losses in a single nursery in southern Ontario due to volutella blight of boxwood exceeded $60,000 (Shi & Hsiang, 2014), and similar economic burdens could be expected across the ornamental industry in other regions.

Despite being one of the most commonly observed diseases affecting boxwood, little is known about the genetics, biology and etiology of the causal agents of volutella blight*.* In this study, we report the first draft genome sequence assembly and annotation of *P. foliicola* and compare the genome characteristics against two other plant pathogenic fungi in the Nectriaceae, *Fusarium graminearum* and *Dactylonectria macrodidyma*. *Fusarium graminearum* is the hemibiotrophic pathogen that causes head blight on wheat and barley, responsible for substantial economic losses in these industries (Goswami & Kistler, 2004). *Dactylonectria macrodidyma* is a destructive necrotrophic pathogen that causes the black foot rot of grapevine and root rots of avocado and olive trees (Urbez-Torres, Peduto & Gubler, 2012; Vitale et al., 2012). Our goal in this study was to compare the genome sequence of *P. foliicola* to these organisms to reveal genome-wide characteristics that may help us better understand the lifestyle of this important fungal pathogen.

## Materials and Methods

### Fungal isolate and nucleic acid isolation

An axenic culture of *P. foliicola* isolate ATCC13545^®^ (also known as isolate A.R. 2711) was used for genome sequencing. This fungal isolate was originally cultured from *B. sempervirens* in Maryland, US. The isolate was grown on potato dextrose agar (BD Difco™, Sparks, MD, USA) for 5-days under 12-h white light photoperiod and then transferred to yeast extract potato dextrose liquid media at 25 °C for 2-days under continuous light. Genomic DNA was extracted from hyphal tissue harvested from liquid media using the OmniPrep DNA kit (G-Biosciences, St. Louis, MO, USA) according to manufacturer’s instructions, and subsequently purified using the Zymo Genomic DNA Clean and Concentrator kit (Zymo Research, Irvine, CA, USA).

### Whole genome sequencing and *de novo* assembly

A genomic DNA library was constructed using the TruSeq Nano DNA Library Prep kit (Illumina, Inc., San Diego, CA, USA) and quantified using the Qubit 2.0 fluorometer (Life Technologies, Grand Island, NY) and the LabChipXT DNA 750 (Caliper Life Sciences, Hopkinton, MA, USA). The library was sequenced on an Illumina MiSeq in two independent runs using paired-end 600-cycle reagent cartridge v.3 (Illumina, Inc.). Reads were processed and assembled using CLC Genomics Workbench v.7.5.1 (CLC Bio, Boston, MA, USA) with a *k*-mer of 24 and a minimum contig length of 500 bp. Illumina adapters were trimmed and low quality reads (Phred score < 0.05) were removed. Summary statistics for the draft genome were generated using CLC Genomics Workbench and QUAST (Gurevich et al., 2013).

Completeness of the *P. foliicola* draft genome assembly was evaluated using BUSCO v.1.1b1 (Simão et al., 2015). The genome assembly of *P. foliicola* used in this study was deposited in NCBI GenBank under accession LMTV00000000, and datasets are also available at the US National Agricultural Library on AgData Commons (http://dx.doi.org/10.15482/USDA.ADC/1408094).

### Nuclear genome annotation

*Ab initio* gene predictions for the draft genome assembly of *P. foliicola* were performed using the MAKER2 v.2.31.6 annotation pipeline (Holt & Yandell, 2011). Gene training was performed after running three rounds of the program according to the program documentation. Gene boundaries were assigned using protein homology evidence from *Fusarium graminearum* strain PH-1 (NCBI BioProject Acc: PRJNA13839; Cuomo et al., 2007). Additional *ab initio* gene predictions were made using the program SNAP (http://korflab.ucdavis.edu/software.html) and AUGUSTUS v.3.2.1 (Stanke et al., 2004) with *F. graminearum* set as the prediction species model organism. New gene predictions using MAKER2 were also performed for the previously published genome assembly of *D. macrodidyma* (Malapi-Wight et al., 2015) using the same parameters as above.

### Identification of transposable elements and repeat-induced mutations

The presence of transposable elements (TEs) was evaluated from the *P. foliicola* and *D. macrodidyma* genomes using the REPET v2.5 (Flutre et al., 2011) pipeline, along with supporting databases Repbase v.20.05 (Kapitonov & Jurka, 2008; Bao, Kojima & Kohany, 2015) and Pfam v.27.0, and run according to the accompanying program documentation (https://urgi.versailles.inra.fr/Tools/REPET). The *TEdenovo* stage was used first to produce a database of four *de novo* identified TEs: an incomplete helitron, a MITE (miniature inverted-repeat TE), a TRIM (terminal-repeat retrotransposon in miniature), and one uncategorized TE sequence. These sequences were then used as initial input for the first of two runs of the *TEannot* pipeline stage, which produced a final genome-wide GFF annotation file using the TE classification scheme described by Wicker et al. (2007). A custom script was used to tabulate counts of TE classes, orders and superfamilies and filtered out fragments less than 80-bp in length according to Wicker et al. (2007) recommendations to avoid misclassification. Results from *TEannot* based on *tblastx* and *blastx* (BLAST+ vr. 2.2.31; Camacho et al., 2009) searches against Repbase were also filtered to remove hits sharing <70% sequence identity.

Individual TE families with ten or more sequences identified, including at least one sequence ≥ 300-bp in length, were checked for signatures of repeat-induced point (RIP) mutation activity using the RIPCAL vr. 2 program (Hane & Oliver, 2008; Hane, 2015). The 300-bp length cutoff was selected based on RIPCAL’s default setting for scanning subsequences and the program was run using both the alignment mode consensus model with ClustalW (Larkin et al., 2007) and di-nucleotide frequency-based methods. Evidence of the signature of RIP mutations was present if di-nucleotide frequencies matched the indexes: (TpA / ApT) ≥ 0.89 and (CpA + TpG) / (ApC + GpT) ≤ 1.03 (Margolin et al., 1998) and visual inspection of RIPCAL alignments showed one or more peaks for (CA ←→TA) + (TG ← →TA) mutations (Hane & Oliver, 2008).

### Mitochondrial genome

The *P. foliicola* and *D. macrodidyma* mitogenomes were identified by performing tBLASTx searches of the complete genome assemblies using the 95.7 kb *F. graminearum* mitochondrial genome as a query (Al-Reedy et al., 2012), with the genetic code set to four (mold mitochondrial). Mitogenomes were annotated using MITOS (Bernt, Donath & Jühling, 2013). Comparative analysis of genes, rRNA and tRNA was performed in the program SimpleSynteny (Veltri, Malapi-Wight & Crouch, 2016) using the *P. foliicola* CDS file to annotate all genomes (e-value cutoff 1e-5, minimum query cutoff 10%), with circular genome mode implementation and genomes organized to minimize Euclidean line distance.

### Identification of mating type idiomorphs

We assessed the presence of the *MAT1-1* and *MAT1-2* idiomorphs in the *P. foliicola* genome by performing a local BLASTn search (e-value cutoff 1e-5) against a *MAT* gene database. The database contained nucleotide sequences for the highly conserved alpha domain DNA binding motif (*MAT1-1-1*) and the high mobility group (HMG) box DNA binding motif (*MAT1-2-1*) for 13 different filamentous fungal species, retrieved from the NCBI GenBank database (Files S1 and S2).

### Phylogenetic reconstruction

A phylogenetic analysis was used to illustrate the relationship between *P. foliicola* and 14 other fungal species. For this analysis, the predicted proteomes of *Aspergillus nidulans* FGSC A4 (ASM114v1; Galagan et al., 2005), *Botrytis cinerea* BcDW1 (Assembly GCA000349525; Blanco-Ulate et al., 2013), *F. graminearum* PH-1 (GCA000240135; Cuomo et al., 2007), *Macrophomina phaseolina* MS6 (GCA000302655; Islam et al., 2012), *Magnaporthe oryzae* 70-15 (MG8; Kim et al., 2010), *Neurospora crassa* (GCA000786625; Baker et al., 2015), *Penicillium oxalicum* 114-2 (GCA000346795; Liu et al., 2013), *Pyrenophora tritici-repentis* (GCA000149985; Manning et al., 2013), *Sclerotinia sclerotiorum* 1980 UF-70 (ASM1469v1; Amselem et al., 2011), *Trichoderma reesei* RUT C-30 (GCA000513815; Koike et al., 2013), *Ustilago maydis* 521 (UM1; Kämper et al., 2006), *Verticillium dahliae* JR2 (GCA000400815; De Jonge et al., 2012) and *Yarrowia lipolytica* CLIB122 (GCA000002525; Dujon et al., 2004) were downloaded from either the EnsemblFungi database (https://fungi.ensembl.org/index.html) or the NCBI Genbank Genome database (https://www.ncbi.nlm.nih.gov/genbank/). The predicted proteome of *D. macrodidyma* generated in our study was also included in the analysis. All proteomes were searched against each other using BLASTp (*e*-value 0.001) and clustered in orthologous gene sets using OrthoMCL v1.4 in the CYVERSE Discovery Environment (https://de.cyverse.org/de/). Single copy genes (clusters with exactly one member per species) found in all 15 fungal proteomes were extracted from the orthologous dataset and the amino acid alignments were performed using MUSCLE v3.8.31 (Edgar, 2004). Gblocks v.0.91b was used to remove ambiguously aligned regions using relaxed selection parameters following Talavera & Castresana (2007). Maximum likelihood (ML) phylogenetic analyses were performed in RAxML (Stamatakis, 2006), using the RAxML GUI v. 1.5b1 (Silvestro & Michalak, 2012). Phylogenetic trees were constructed using the JTT matrix-based model (Jones, Taylor & Thornton, 1992) and 1,000 bootstrap replicates. *Ustilago maydis* 521 was used as outgroup in the phylogenetic analyses.

### Estimation of evolutionary divergence times

To obtain approximate information on divergence events, the phylogenetic tree was timed using RelTime (Tamura et al., 2012) as implemented in MEGA 7 (Kumar, Stecher & Tamura, 2016). RelTime estimated the relative divergence times for each node of the ML tree using the same outgroup taxa. We used seven confidence intervals obtained from the TimeTree database (http://timetree.org; Hedges et al., 2015) as minimum and maximum times to convert the relative times into absolute times (Table S4). Time estimates were performed using maximum-likelihood branch length, local clocks, a JTT matrix-based model (Jones, Taylor & Thornton, 1992) and a discrete Gamma distribution among sites (five categories).

### Comparative genomic analyses

The genome sequences of *D. macrodidyma* JAC15-245 (NCBI GenBank accession JYGD00000000) and *F. graminearum* PH-1 were downloaded from NCBI GenBank and Ensembl-Fungi database respectively, and used for comparative analysis against the *P. foliicola* draft genome assembly generated in this study. The predicted proteome generated from this study was used for *D. macrodidyma,* while the published proteome dataset for *F. graminearum* was downloaded from the Ensembl-Fungi database. Genome-wide identification, comparison and visualization of orthologous gene clusters among *P. foliicola*, *D. macrodidyma* and *F. graminearum* were performed using the web platform OrthoVenn (http://www.bioinfogenome.net/OrthoVenn/; Wang et al., 2015). The program uses a modified OrthoMCL algorithm to identify orthologous gene clusters from the UniProt/Swiss-Prot database. Proteins potentially involved in carbohydrate metabolism were annotated for each proteome by searching against the database of automated Carbohydrate-active enzyme ANnotation (dbCAN; http://csbl.bmb.uga.edu/dbCAN/annotate.php; Yin et al., 2012). A *X*^2^ test of independence, similar to that used in Martinez et al. (2008), was used to identify statistically significant differences across the CAZyme repertoire. Identification of putative enzymes related to the biosynthesis of secondary metabolites was performed from the predicted proteins using the web-based program AntiSMASH (http://antismash.secondarymetabolites.org; Medema et al., 2011; Weber et al., 2015). The secretome was predicted by screening the predicted proteins for different features using a bundle of eight different prediction tools implemented in the web-based program SECRETOOL (Cortázar et al., 2014). Pathogenicity associated genes were identified by performing a local BLASTp search of the predicted proteome against the curated pathogen-host interaction database (PHI-base v.4.0; http://www-phi4.phibase.org/; Winnenburg et al., 2008).

## Results

### Nuclear genome assembly and annotation

A *de novo* genome assembly of *P. foliicola* ATCC13545^®^ was generated from 19.5 million paired end, 300-bp reads, comprising a total of 5.4 Gb of raw sequence data. The resulting genome assembly for *P. foliicola* was 28.7 Mb, organized in 425 scaffolds (≥500-bp), with an average read depth coverage of 188-fold (Table 1). The N50 scaffold length is 184,987 bp and the three longest scaffolds are 619,696 bp, 551,775 bp, and 524,658 bp. Analysis of the *P. foliicola* genome assembly using BUSCO identified 420 complete genes out of 429 conserved eukaryotic ortholog dataset and 1,416 complete genes out of 1,438 conserved fungal ortholog dataset (genome completeness scores of 97% and 98%, respectively). *Ab initio* gene predictions performed with the MAKER2 pipeline for the *P. foliicola* genome assembly identified 9,272 protein-coding genes with an average predicted protein length of 484 amino acids (Table 1). Meanwhile, gene predictions for the genome of *D. macrodidyma* identified 16,959 protein-coding genes with an average length of 478 amino acids. Of the three genomes evaluated in this study, gene number predictions were highest for *D. macrodidyma*, which also had the largest estimated genome size at 58 Mb when compared to *P. foliicola* and *F. graminearum* (13,313 genes, 36.1 Mb; Cuomo et al., 2007).

**Table 1:** Genome assembly and annotation statistics for *Pseudonectria foliicola*, *Dactylonectria macrodidyma*, and *Fusarium graminearum*. Values are given in base pairs, unless otherwise specified.

**Genome features**	***Pseudonectria foliicola***	***Fusarium graminearum***	***Dactylonectria macrodidyma***
Genome size (Mb)	28.7	38.1	58.0
Average sequence coverage	188	10	46
Total number of scaffolds	425	5	850
GC content (%)	54.3	48.2	49.9
Transposable elements (%)	0.7	0.06	6.5
Predicted proteins	9,272	14,164	16,454
NCBI accession	LMTV00000000	AACM00000000	JYGD00000000

### Transposable elements, repetitive DNA and repeat induced mutations

Only 0.7% (196,205-bp) of the *P. foliicola* nuclear genome assembly contained TEs based on the REPET pipeline analysis (Table 1). Annotation of TEs across the *P. foliicola* genome assembly identified 191 TE matches. Of these, 141 were Class I (retrotransposons) TEs comprising ∼0.4% of genome and 45 were Class II (DNA) TEs making up ∼0.3% of the genome. Long terminal-repeats (LTRs) were found to be the most abundant Class I order (∼0.3% of genome) and included matches in the Copia, Gypsy and BEL/Pao retrotransposon superfamilies. Almost all Class II TEs were identified as Helitrons, with only ∼0.001% of the genome identified with TIR or “unknown” TEs (Wicker code: DXX). Evidence of RIP mutation affecting *P. foliicola* TEs was only found for Helitrons based on alignment of 37 sequences (3.7-kb in length) and di-nucleotide indexes: TpA/ApT = 1.8 and (CpA + TpG)/(ApC + GpT) = 0.3.

The draft genome of *D. macrodidyma* genome as originally published did not include information about TEs. Here, we also analyzed that assembly for the presence of TEs and RIP mutations using the same parameters used for *P. foliicola* for comparative purposes. As REPET identified over 2,000 *D. macrodidyma* TEs of “unknown” superfamily, we performed an additional *tblastx* search (e-value: 0.01, percent query coverage per hsp: ≥70%, minimum percent identity of hits: ≥70%) against Repbase and reclassified five TEs based on the top match if it shared the same class and order. REPET analysis identified ∼6.5% (3,794,887-bp) of the *D. macrodidyma* genome to be made up of TEs (2,178 elements). Of these, 1,489 elements were Class I TEs and comprised ∼5.3% of the genome and 689 were Class II TEs and comprised ∼1.2% of the genome. The most common Class I retrotransposon orders were: LTRs (∼3.2%), “Unknown RXX” (∼1.1%), DIRS-like (∼0.9%), LINE (∼0.1%) and SINE (0.001%). For Class II DNA elements, the most common orders were: TIR (∼0.7%), Helitron (∼0.4%) and “Unknown DXX” (∼0.2%). Evidence for RIP mutation affecting *D. macrodidyma* TEs using alignment and di-nucleotide counts was identified in the sets of 91 Helitron and 146 DIRS TEs. For the Helitrons, RIP indexes were calculated as: TpA/ApT = 1.3 and (CpA + TpG)/(ApC + GpT) = 0.9, while for DIRS: TpA/ApT = 1.4 and (CpA + TpG)/(ApC + GpT) = 0.3.

### Mitochondrial genome size and gene content

The mitogenomes of *P. foliicola* and *D. macrodidyma* were each contained within a single scaffold, measuring 58.3 kb and 44.2 kb respectively (scaffold 56, scaffold 68). Although the mitogenome-containing scaffolds for these two organisms were considerably smaller than that of *F. graminearum* (95.7 kb), a full complement of protein-coding genes was contained: apocytochrome b (*cob*), ATP-synthase subunits (*atp6, atp8, atp9*), cytochrome oxidase subunits (*cox1, cox2, cox3*), and NADH subunits (*nad1, nad2, nad3, nad4, nad4L, nad5, nad6*). This set of 14 protein-coding mitochondrial genes is highly conserved among fungi, and shared with animal mtDNA (Bullerwell & Lang, 2005). A full complement of tRNAs necessary for translation was encoded in the mitogenomes (25 total), as were small and large subunit rRNAs (one and three copies, respectively). Neither *P. foliicola* nor *D. macrodidyma* mitogenomes contained copies of the four large unidentified open-reading frames that are encoded by the *F. graminearum* mitogenome (Al-Reedy et al., 2012), accounting for the observed difference in sizes between these species.

#### Mitochondrial genome synteny

**Figure 2:**
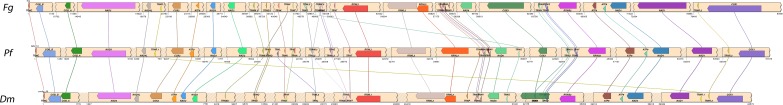
Comparisons of the mitogenomes of *Pseudonectria foliicola (Pf), Dactylonectria macrodidyma (Dm)* and *Fusarium graminearum (Fg)*. Shared areas contain intergenic sequence that has been collapsed to improve diagram readability; the starting and ending coordinates are plotted at the beginning and end of these regions.

The organization of the mitogenomes of *P. foliicola*, *D. macrodidyma* and *F. graminearum* was highly conserved at the gene level. As observed in the mitochondria of most ascomycete fungi, all genes, rRNAs and tRNAs were oriented in the same direction. The three mitogenomes contained a nearly identical ordering of shared genes (Fig. 2). Only a single tRNA (tRNA-G) was positioned differently in the *P. foliicola* and *D. macrodidyma* mitogenomes relative to *F. graminearum*. Two tRNAs (tRNA-R and tRNA-L) were positioned differently between *D. macrodidyma* and *P. foliicola*/*F. graminearum*. This conserved ordering of the three mitogenomes was consistent with previous observations that the mitochondrial DNA of fungi in the order Hypocreales is highly conserved (Al-Reedy et al., 2012; Pantou, Kouvelis & Typas, 2008).

### Identification of mating type idiomorphs in *P. foliicola*

The presence of the conserved alpha domain indicative of the *MAT1-1* mating type idiomorph was identified in *P. foliicola* scaffold 102. In the same scaffold, the *APN2* (encoding DNA lyase) and *SLA2* (encoding cytoskeletal protein) genes were found flanking the mating type idiomorph, as well as the MAT-locus associated gene *COX13* (cytochrome c oxidase subunit VIa homolog). The HMG-box region indicative of the *MAT1-2* mating type idiomorph was not found within the *P. foliicola* genome. Based on these data, *P. foliicola* appears to be a heterothallic fungus, requiring a partner of the alternate mating type in order to initiate the sexual life cycle.

### Phylogeny and divergence estimation

**Figure 3:**
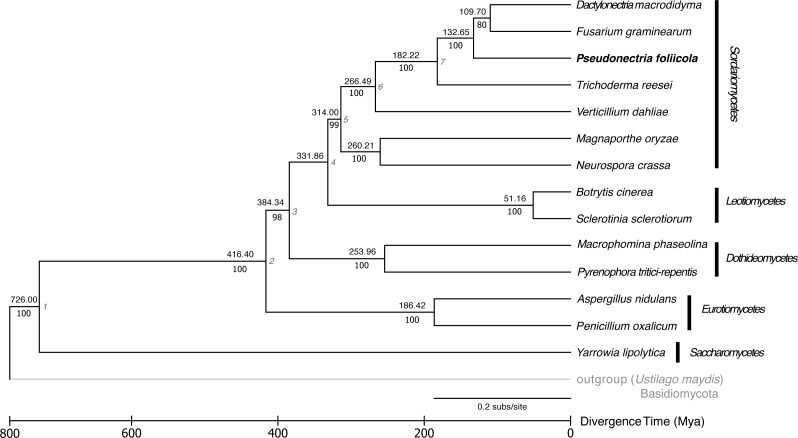
Reconstruction of the phylogenetic relationships and divergence times of *Pseudonectria foliicola* relative to other fungal species. The maximum likelihood (ML) tree analysis and time tree (RelTime method) were conducted on the concatenated dataset of 1,511 single copy orthologous genes. Numbers above branches indicate the approximate relative times of divergence (Mya) between two lineages. The seven calibration points used are indicated at the nodes and listed in Table S4. Scale representation under the tree demonstrate divergence times of genes. Statistical support values corresponding to ML are indicated below the branches. The basidiomycete *Ustilago maydis* was used as the outgroup.

The phylogenetic relationships and divergence times among the fungal genomes studied is displayed in Fig. 3. Fourteen publicly available fungal genomes were used to examine the phylogenetic placement of *P. foliicola* through the analysis of single copy orthologous genes. The program OrthoMCL identified 16,356 gene clusters, from which 1,884 orthologous genes were shared across all 15 fungal species. From these shared gene clusters, 1,511 orthologous genes present as single copies were used for the phylogenetic analysis. The final dataset after removal of ambiguously aligned regions consisted of 388.7 Mb. The ML analysis identified, with high bootstrap support (>70%), five major clusters representing the ascomycete classes Sordariomycetes, Leotiomycetes, Eurotiomycetes, Dothideomycetes, and Saccharomycetes (Fig. 3). Within the Sordariomycetes, *P. foliicola* was more closely related, albeit appearing basal, to *D. macrodidyma* and *F. graminearum*, all of which belonged to the Nectriaceae in the order Hypocreales. These phylogenetic relationships were consistent with previous reports (Lombard et al., 2015; Yin et al., 2015). Using RelTime methods and a JTT matrix-based model, the estimated log likelihood value was −142633.0660. The divergence of *P. foliicola* from the Nectriaceae species *D. macrodidyma* and *F. graminearum* was estimated to have occurred ∼132 Mya. The overall estimates of divergence times in the tree are in agreement with divergence times reported for the order Hypocreales (Sung, Poinar & Spatafora, 2008).

### Comparative genomic analysis of *P. foliicola* and other fungi in the Nectriaceae

#### Gene orthology

**Figure 4:**
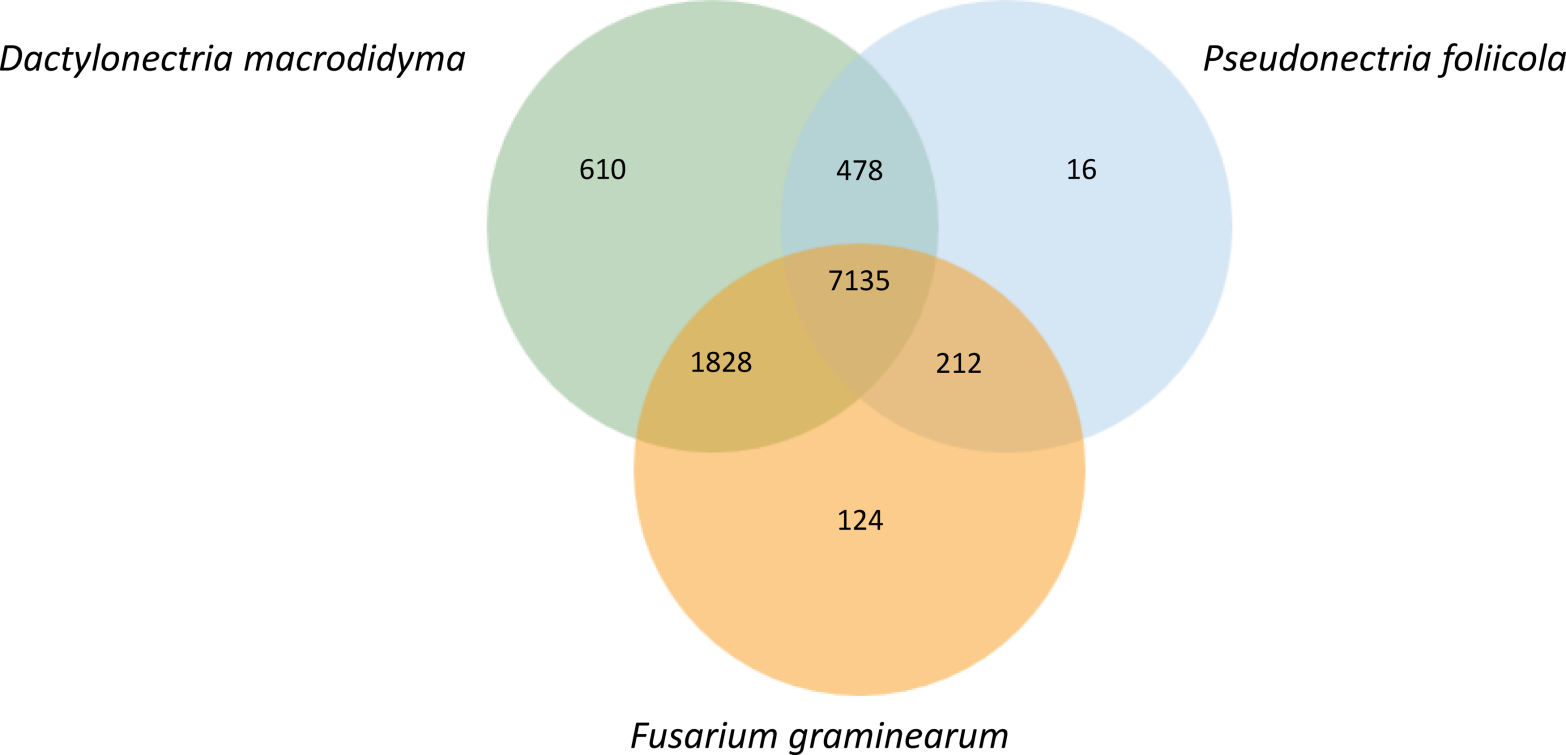
Orthologous genes shared between *Pseudonectria foliicola*, *Dactylonectria macrodidyma* and *Fusarium graminearum*.

Orthologous gene clusters were identified for *P. foliicola*, *D. macrodidyma* and *F. graminearum* using OrthoVenn (Fig. 4). Proteins from these three fungal species formed 10,403 orthologous clusters, of which 7,135 clusters were shared among all three species. The top three Swiss-Prot annotations among the core shared clusters were the Acyl-CoA-binding domain-containing protein (44 proteins), pleiotropic drug resistance protein 4 (30 proteins) and a short-chain dehydrogenase TIC 32, chloroplastic (12 proteins). Sixteen clusters representing 48 predicted proteins were unique to *P. foliicola*, while 610 and 124 species-specific protein clusters were identified in *D. macrodidyma* and *F. graminearum*, respectively. Only 0.2% of the *P. foliicola* proteome was unique, a low percentage relative to 1.3% for the *F. graminearum* and 6.0% for the *D. macrodydima* proteomes. Although the majority of the 16 clusters unique to *P. foliicola* had no annotations based on the UniProt/Swiss-Prot and Gene Ontology (GO) databases, three of the unique clusters were identified as (1) vegetative cell wall protein Gp1/structural constituent of cell wall (GO:0005199), (2) thioredoxin/protein disulfide oxidoreductase activity (GO:0009507), and (3) leucine-rich repeat extensin-like protein 3/structural constituent of cell wall (GO:0005618). Biological processes and molecular functions annotated by the GO database for the species-unique gene clusters were most abundant in *D. macrodidyma* (66 biological processes, 28 molecular functions), and least abundant in *P. foliicola* (five biological processes, two molecular functions; Table S1). Three GO categories were found enriched (hypergeometric test on OrthoVenn, *p*-value <0.05) in the *P. foliicola* unique clusters: (1) a glycerol ether metabolic process, (2) structural constituent of cell wall and, (3) a protein disulfide oxidoreductase activity. Despite the large number of unique gene clusters found in *D. macrodidyma*, only one GO category, an oxidoreductase activity acting on single donors with incorporation of molecular oxygen, was found to be enriched.

#### The CAZyme repertoire

**Figure 5:**
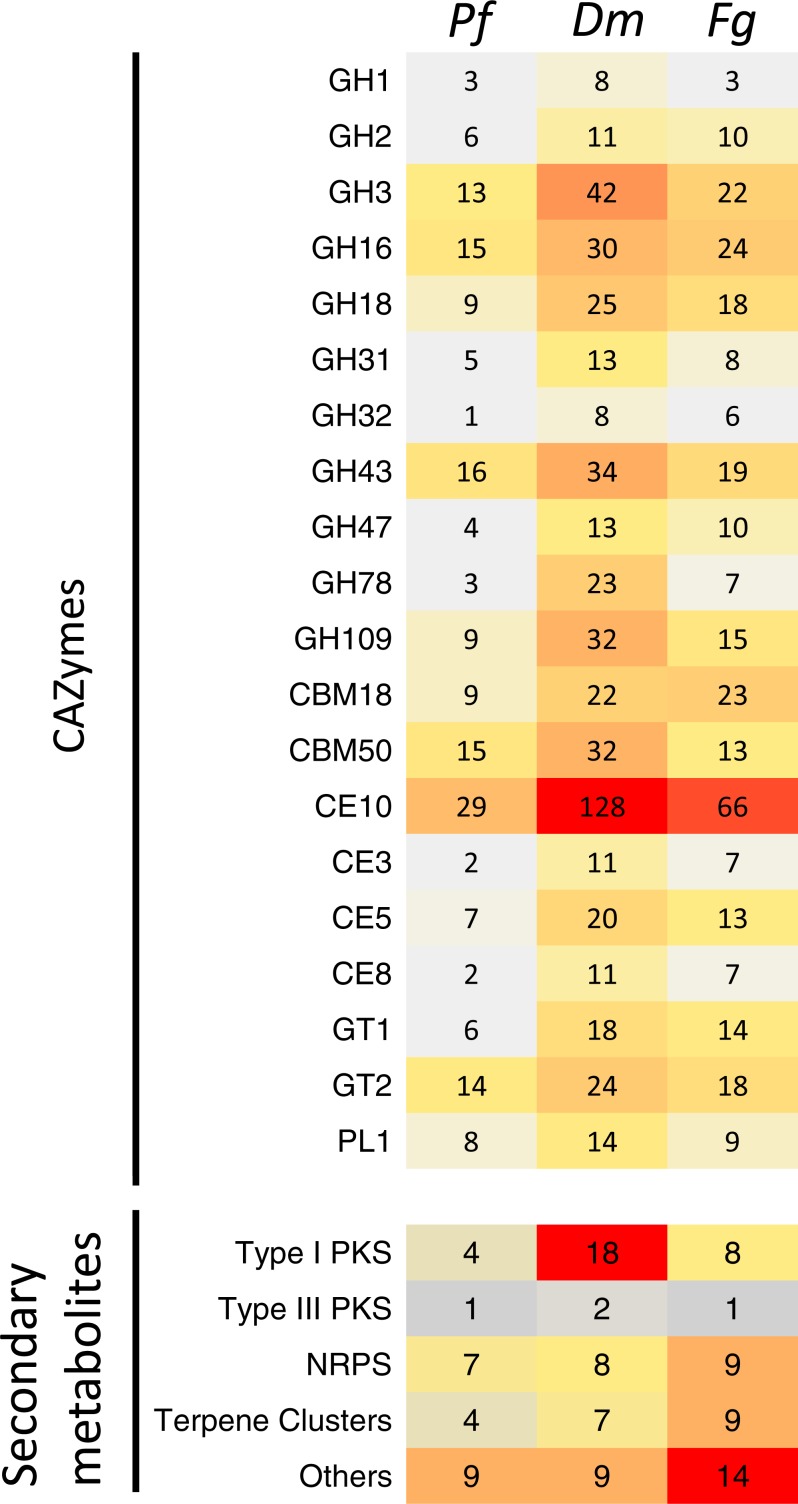
Comparison of the predicted CAZymes and secondary metabolite clusters identified from the genome assemblies of *Pseudonectria foliicola (Pf)*, * Dactylonectria macrodidyma(Dm)* and *Fusarium graminearum (Fg)*. GH, glycoside hydrolases; CBM, Carbohydrate-binding modules; CE, Carbohydrate esterases; GT, Glycosyl transferases; PL, Polysaccharide lyases; PKS, Polyketides; NRPS, Non-ribosomal peptide synthase.

Annotation of the *P. foliicola* proteome identified 448 CAZyme modules, including domains encoding 179 glycoside hydrolases (GH), 88 glycosyl-transferases (GT), 18 polysaccharide lyases (PL), 64 carbohydrate esterases (CE), 46 carbohydrate-binding modules (CBM), and 53 enzymes with auxiliary activities (AA) (summarized in Table 2, Fig. 5 and Table S2). The *D. macrodidyma* and *F. graminearum* genomes encoded 1,086 and 707 CAZyme modules, respectively. The CAZyme encoding genes represented between 4.8 and 6.6% of the predicted proteome for the three fungal species (Table 2).

**Table 2:** Summary of the carbohydrate-active enzyme (CAZyme) modules identified from the predicted proteome of *Pseudonectria foliicola*, *Dactylonectria macrodidyma* and *Fusarium graminearum*. RF%: Relative frequency of CAZyme modules over the total number of predicted proteins for the corresponding genome.

**Name**	*Pseudonectria foliicola*	**RF**%	*Dactylonectria macrodidyma*	**RF**%	*Fusarium graminearum*	RF%
AA	53	0.52	147	0.84	110	0.79
CBM	46	0.53	116	0.73	80	0.65
CE	64	0.83	210	1.37	127	1.08
GH	179	1.94	447	2.59	268	2.01
GT	88	1.01	125	0.78	100	0.82
PL	18	0.17	41	0.20	22	0.16
**Total**	448	4.83%	1,086	6.40%	707	5.31%

**Notes.**

AA Auxiliary activity families CBM Carbohydrate-binding modules CE Carbohydrate esterase families GH Glycoside hydrolase families GT Glycosyltransferase families PL Polysaccharide lyase families

An increased number of CAZyme modules was identified from the *D. macrodidyma* proteome when compared to *P. foliicola*, *F. graminearum* and a database of 91 other plant pathogenic, facultative pathogenic, saprophytic and symbiotic fungi (Table S2; Zhao et al., 2013). Although not significant in *X*^2^ tests against *P. foliicola* and *F. graminearum*, *D. macrodidyma* has an increased number of GH and CE enzymes. This is largely due to the GH3 family, which is associated with cellulose degrading activities, the GH28 family, with pectinase activities, as well as the less characterized GH78 and GH109 families. Within the CE and PL module groups, increased numbers in the *D. macrodidyma* genome were observed in the families CE3, CE5, CE10 and PL1 (Fig. 5). The CE3 and CE5 have acetyl xylan esterase and cutinase activities and are common modules with a higher representation in Ascomycetes relative to Basidiomycetes (Zhao et al., 2013). CE10 families have carboxylesterases activities but can also act on non-carbohydrate substrates (Cantarel et al., 2009). The PL1 family is the most commonly found among fungi, particularly in plant pathogenic species (Zhao et al., 2013). The number of CE families in *D. macrodidyma* (233) is comparable to that of the pea root pathogen *Fusarium solani*, previously regarded as having the most CEs with 223 (Zhao et al., 2013).

#### Secondary metabolite clusters

Annotation of the *P. foliicola*, *F. graminearum* and *D. macrodidyma* proteomes identified key enzyme clusters for the biosynthesis of secondary metabolites such as non-ribosomal peptide synthases (NRPS), polyketide synthases (PKS), terpene synthases (TS), among others (summarized in Fig. 5). The genome of *P. foliicola* contained 25 secondary metabolite clusters, in contrast with *F. graminearum* and *D. macrodidyma* with a total of 41 and 44 clusters, respectively (Fig. 5).

#### Secretome

The predicted secretome for *P. foliicola* was also relatively small, comprising just 346 proteins. In comparison, the genomes of *D. macrodidyma* and *F. graminearum* contained 607 and 457 predicted secreted proteins (Table S3 ). However, for all three species, the secretome made up between 3.4 to 3.7% of the predicted proteome.

#### Virulence associated genes

The genomes of *P. foliicola*, *D. macrodidyma* and *F. graminearum* were screened against PHI-base, a curated database that contains pathogenicity, virulence and effector genes from fungi, oomycete and bacterial pathogens (Winnenburg et al., 2008). Relative to the total proteome, the frequency of virulence-associated genes was highest in *P. foliicola* (14.5%) compared to *F. graminearum* (12.2%) and *D. macrodidyma* (9.5%) (Table 3). Genes associated with the loss of pathogenicity, reduced virulence and those with mixed outcomes were identified in higher frequencies in the *P. foliicola* proteome than in the *D. macrodidyma* and *F. graminearum* proteomes (Table 3). Genes associated with loss of pathogenicity and with reduced virulence have been identified by PHI-base from transgenic strains of fungal, oomycete and bacterial pathogens that either fail to cause disease or that cause quantitatively lower degrees of disease than the wild-type strains (Winnenburg et al., 2008; Urban et al., 2015).

**Table 3:** Summary of the predicted genes associated with virulence in the genome assemblies of *Pseudonectria foliicola*, *Dactylonectria macrodidyma* and *Fusarium graminearum*.

**Name**	***Pseudonectria foliicola***	**RF**%	***Dactylonectria macrodidyma***	**RF**%	***Fusarium graminearum***	**RF**%
Chemistry target	7	0.08	8	0.05	9	0.07
Effector	15	0.16	22	0.13	19	0.14
Enhanced antagonism	2	0.02	2	0.01	2	0.02
Increased virulence	7	0.08	7	0.04	7	0.05
Increased virulence (Hypervirulence)	13	0.14	20	0.12	18	0.14
Lethal	65	0.70	80	0.47	87	0.65
Loss of pathogenicity	103	1.11	116	0.68	99	0.74
Mixed outcome	112	1.21	124	0.73	114	0.86
Reduced virulence	459	4.95	510	3.01	490	3.68
Unaffected pathogenicity	558	6.02	712	4.2	782	5.87
Other	2	0.02	2	0.01	2	0.02
**Total**	**1,343**	**14.5%**	**1,603**	**9.45%**	**1,629**	**12.24%**

## Discussion

*Pseudonectria* species, *P. foliicola* and *P. buxi*, are economically important fungal pathogens responsible for increased costs in foliar disease management of boxwood plants worldwide. Here, we present a draft genome sequence for *P. foliicola*, including a comparative analysis of this genome against two other plant pathogens in the Nectriaceae. The 28.7 Mb *P. foliicola* draft genome assembly is smaller than the reported size of other fungi in the Ascomycota (average genome size 36.9 Mb; Mohanta & Bae, 2015). This assembly represents the smallest genome known from the Nectriaceae, in which genomes range from 36.1 to 58.1 Mb: *D. macrodidyma* (58.0 Mb; Malapi-Wight et al., 2015), *Neonectria ditissima* (44.9 Mb; Gomez-Cortecero, Harrison & Armitage, 2015) and *F. graminearum* (36.1 Mb; Cuomo et al., 2007). Consistent with genome size, the number of predicted gene models from the *P. foliicola* assembly is reduced but comparable to the number of gene models predicted in other Ascomycota fungi with similar genome size (e.g., *Patellaria atrata*, 28.7 Mb, 7,794 gene models (JGI); Mohanta & Bae, 2015). *Pseudonectria foliicola* also contains one of the smallest cohorts of TEs reported for filamentous fungi, similar to the genomes of *Trichoderma atroviridae*, *T. reesei*, and *T. virens* (ranging from 0.48 to 0.57%; Kubicek et al., 2011; Martinez et al., 2008) as well as *F. graminearum* (<1% of repetitive DNA; Cuomo et al., 2007). A strong correlation between genome size and repeat content was reported in a study of 18 Dothidiomycete genomes (Ohm et al., 2012) however, based on the low percentage of TEs found in *F. graminearum* and *D. macrodidyma*, this correlation may not be sustained within the Nectriaceae.

The uniquely small genome size of *P. foliicola* is similar to the genome assembly recently reported from *Escovopsis weberi* (29.5 Mb), a highly specialized mycoparasitic fungus that also belongs to the order Hypocreales (De Man et al., 2016). Specialized pathogens or those with a narrow host range are predicted to maintain only the essential cohort of genes, and lose those no longer needed in their particular niche (e.g., De Man et al., 2016; Lee & Marx, 2012) and while there are exceptions (e.g., Spanu et al., 2010), the comparative genome analysis of the three Nectriaceae genomes in this study follows that prediction. From the three fungal species analyzed in this study, only *P. foliicola* has a narrow host range (only known from *Buxus* spp.) while *F. graminearum* (reported frequently in a large number of hosts in Poaceae) and *D. macrodidyma* (reported on grapevine, avocado and olive trees) have increasingly broader host ranges and larger genomes and proteomes. Divergence time estimates indicated that *P. foliicola*, *D. macrodidyma* and *F. graminearum* diverged from their common ancestral organism ca. 132 Mya. This relatively distant split may account for the differences observed in pathogenicity and host range between these species, and indicate that ongoing gene loss resulting in a reduced genome size is a major contributor to the genome evolution of *P. foliicola*.

Enzymes that degrade plant cell wall carbohydrates can be essential during the infection and decomposition of host plant tissue, particularly for necrotrophic and hemibiotrophic fungi (Gibson et al., 2011; Zhao et al., 2013). Consequently, CAZyme profiles can be used as indicators of the fungal lifestyle. In previous studies, necrotrophic and hemibiotrophic fungal plant pathogens have been reported to produce a large repertoire of these enzymes (Gibson et al., 2011; Knogge, 1996), and typically exhibit expanded arsenals of CAZymes in their genomes, relative to biotrophic and obligate fungi that typically exhibit the lowest numbers (Zhao et al., 2013). Similarly, the fungal secretome and secondary metabolites, both involved in the host-pathogen interaction process (Van den Burg et al., 2006; Yu & Keller, 2005), can correlate with the lifestyle of a fungal pathogen (Lowe & Howlett, 2012; Ohm et al., 2012). Consistent with its small genome and proteome size, *P. foliicola* has the smallest cohort of CAZymes, SM clusters and secreted proteins relative to *D. macrodidyma* and *F. graminearum*. Despite the reduced number of total CAZyme clusters in *P. foliicola*, all clusters are well represented and comparable to those found in the genomes of fungi with different lifestyles (comparison among the 91 fungal CAZyme profiles by Zhao et al., 2013). Within the Nectriaceae, comparisons among *P. foliicola*, *D. macrodidyma* and *F. graminearum* show increased numbers of CAZymes in several clusters for *D. macrodidyma* relative to the other two fungi, although not significant. The glycosyl hydrolases (GH), enzymes with an important role in the complete breakdown of the plant cell wall for successful infection (Cantarel et al., 2009), were the most abundant type of secreted protein and CAZymes found across all three species compared.

The pathogenicity profile of *P. foliicola* as predicted by comparisons against the PHI-base database shows a higher relative frequency of genes associated with loss of pathogenicity and reduced virulence, when compared to *D. macrodidyma* and *F. graminearum*. The genome characteristics of *P. foliicola* described in our analyses may help explain the apparent inability of this fungus to penetrate host plant tissue and its dependence on wounding or winter damage for successful infections. Shi & Hsiang (2014) reported primary infection by *P. buxi* on leaves and stems of various *Buxus* species through wounded tissue resulting in general plant decline. A similar strategy may be employed by the closely related species *P. foliicola*; however, due to its recent taxonomic placement, it remains uncertain whether previous disease reports and epidemiology studies correspond to either *Pseudonectria* species.

## Conclusions

Despite the economic importance of fungi in the Nectriaceae family, only a small number of genome resources are currently available. A survey of public databases shows that less than 5% of the estimated 900 fungal species in this family have been sequenced on the whole genome scale (NCBI-GenBank, the Joint Genome Institute Mycocosm and Ensembl databanks). To our knowledge, the *P. foliicola* genome is the smallest known genome in the Nectriaceae. Currently, it is unknown if the genome characteristics of other fungal pathogens in the Nectriaceae are similar to those of *P. foliicola*, *D. macrodidyma* or *F. graminearum*, or if these genomes represent the extremes. With the advent and accessibility of next generation sequencing technologies we expect that more in depth comparative genomics studies will characterize fungal groups of great economic and ecological importance. The quality of microbial draft genomes and consequently the predicted size of the associated proteome can also be influenced by the next generation sequencing platform and assembly software (Mavromatis et al., 2012). Even though improvements on sequencing chemistry and better assembly algorithms have reduced the chance of errors, further sampling of genomes of fungi in the Nectriaceae and other families, ideally with a wide range of life styles, would help determine if the differences observed in annotation rates for some protein classes in our study is due to real biological differences or if they might be an artifact of the technology used to generate these draft genomes. Furthermore, the availability of fungal genomes will aid in the resolution of important fungal lineages and explore beyond the commonly used standard molecular markers for taxonomic classification.

##  Supplemental Information

10.7717/peerj.5401/supp-1 File S1 MAT1-1 database

10.7717/peerj.5401/supp-2 File S2 MAT1-2 database

10.7717/peerj.5401/supp-3 Data S1 RaxML file, TimeTree, and alignments

10.7717/peerj.5401/supp-4 Table S1 Molecular functions, biological processes, and cellular components annotated by the Gene Ontology (GO) database using OrthoVenn (http://www.bioinfogenome.net/OrthoVenn/, on November 22, 2017)

10.7717/peerj.5401/supp-5 Table S2 Comparative analysis of fungal CAZymes (from Zhao et al., 2013 and this study) CBM, carbohydrate binding module; CE, carbohydrate esterase; GH, glycoside hydrolases; GT, glycosyltransferase; PL, polysaccharide lyase.

10.7717/peerj.5401/supp-6 Table S3 Predicted secretome for Pseudonectria foliicola, Fusarium graminearum, and Dactylonectria macrodidyma

10.7717/peerj.5401/supp-7 Table S4 Calibration points matching the taxa used in the phylogenetic tree obtained from treetime.org

## Additional Information and Declarations

### DNA Deposition

The following information was supplied regarding the deposition of DNA sequences: The genome assembly of *P. foliicola* used in this study was deposited in NCBI GenBank under accession LMTV00000000, and datasets are also curated by the US National Agricultural Library on AgData Commons (http://dx.doi.org/10.15482/USDA.ADC/1408094).

### Data Availability

The following information was supplied regarding data availability: US National Agricultural Library on AgData Commons
http://dx.doi.org/10.15482/USDA.ADC/1408094. This data are also provided in the Supplemental Files.
